# Morphological characterization and transcriptome analysis of rolled and narrow leaf mutant in soybean

**DOI:** 10.1186/s12870-024-05389-7

**Published:** 2024-07-19

**Authors:** Xiaomin Xu, Yongzhen Wang, Housheng Lu, Xueqian Zhao, Jiacan Jiang, Mengshi Liu, Cunyi Yang

**Affiliations:** 1 Guangdong Provincial Key Laboratory of Plant Molecular Breeding, College of Agriculture, South China Agricultural University, Guangzhou, 510642 China; 2 Key Laboratory for Enhancing Resource Use Efficiency of Crops in South China, Ministry of Agriculture and Rural Affairs, South China Agricultural University, Guangzhou, 510642 China

**Keywords:** Rolled and narrow leaf, Leaf type, Cytokinin, Soybean

## Abstract

**Background:**

In plants, the leaf functions as a solar panel, where photosynthesis converts carbon dioxide and water into carbohydrates and oxygen. In soybean, leaf type traits, including leaf shape, leaf area, leaf width, and leaf width so on, are considered to be associated with yield. In this study, we performed morphological characterization, transcriptome analysis, and endogenous hormone analysis of a rolled and narrow leaf mutant line (*rl*) in soybean.

**Results:**

Compared with wild type HX3, mutant line *rl* showed rolled and narrower leaflet, and smaller leaf, meanwhile *rl* also performed narrower pod and narrower seed. Anatomical analysis of leaflet demonstrated that cell area of upper epidermis was bigger than the cell area of lower epidermis in *rl*, which may lead rolled and narrow leaf. Transcriptome analysis revealed that several cytokinin oxidase/dehydrogenase (CKX) genes (*Glyma.06G028900*, *Glyma.09G225400*, *Glyma.13G104700*, *Glyma.14G099000*, and *Glyma.17G054500*) were up-regulation dramatically, which may cause lower cytokinin level in *rl*. Endogenous hormone analysis verified that cytokinin content of *rl* was lower. Hormone treatment results indicated that 6-BA rescued rolled leaf enough, rescued partly narrow leaf. And after 6-BA treatment, the cell area was similar between upper epidermis and lower epidermis in *rl*. Although IAA content and ABA content were reduced in *rl*, but exogenous IAA and ABA didn’t affect leaf type of HX3 and *rl*.

**Conclusions:**

Our results suggest abnormal cytokinin metabolism caused rolled and narrow leaf in *rl*, and provide valuable clues for further understanding the mechanisms underlying leaf development in soybean.

**Supplementary Information:**

The online version contains supplementary material available at 10.1186/s12870-024-05389-7.

## Introduction

Soybean (*Glycine max* (L.) Merr) is an important crop, which is crucial source of vegetable protein and animal feed. Continual increases in soybean yield is critical to meet global demands. Leaf is the main organ of photosynthesis for soybean, meanwhile, leaf morphological also is associated with canopy and is the key determinant of light interception and distribution dynamics [1–4]. Leaf type traits, such as leaf size, leaf shape, and leaf distribution, are associated with yield, because leaf traits not only affect photosynthetic efficiency, but also affect planting density [5–9]. Several researches have supported that leaf type has significant effect on light environments of canopy and soybean yield [10]. Thus, the study of leaf development is essential for improving soybean yield.

Leaf development is a complicated process, complex genetic networks regulate leaf morphogenesis together [11, 12]. Soybean leaf shape mainly includes broad leaflet and narrow leaflet. So far, a number of QTLs about leaf shape in soybean had been reported [13–18], among them, a few genes were cloned. *Ln* was mapped by RILs on chromosome 20, encoded a protein which contained EAR motif, and was homologous to *Arabidopsis JAGGED* (*JAG*). The transition from broad leaflet (*Ln*) to narrow leaflet (*ln*) was associated with an amino acid substitution in the EAR motif. Furthermore, *ln* could increase number of seeds per pod [19, 20]. *GmCTP* was mapped using mutant on chromosome 05, and could regulate many key regulators in leaf development. *ctp* leaded to chicken toes-like leaf [17]. For leaflet area, GmSIZ_1_a and GmSIZ_1b_ are small ubiquitin-related modifier (SUMO) E3 ligase, and positively regulate vegetable growth through mediating SUMO modification. *GmSIZ*_*1*_ *RNAi* plants showed smaller leaf area [21]. In contrast, CRISPR/Cas9-induced mutation in *GmKIX8-1* resulted in larger leaflet size and seed size by increasing cell proliferation [22].

Hormones also play an important role in the formation process of leaf. Leaf begins as a simple primordium at the shoot apical meristem (SAM). Leaf initiation activity is intimate connected to the auxin. The accumulation of auxin is created by PIN which is the efflux transporter with polar localization at defined points [23, 24]. Auxin maxima could reduce the expression of KNOX in the presumptive region of leaf primordia in the SAM, which results in the initiation of leaf primordia [25]. In SAM, the KNOX transcription factors express, positively regulate cytokinin synthesis and keep their high level [26]. Then, cytokinin promote the expression of WUS, which maintain a high cell division rate in SAM [27, 28]. Therefore, the leaf initiation at the SAM is controlled by auxin and cytokinin together. What’s more, auxin/cytokinin ratio could change the initiation of lateral organs, which may lead phyllotactic shift [29]. For leaf growth, auxin influences leaf size through cell proliferation and cell expansion. Auxin homeostasis and signaling are disordered that lead to smaller leaf size [30, 31]. When GA synthesis or signaling is abnormal, leaf size is reduced [32]; while, over-expressing *GA20ox1* produce larger leaf [33]. Thus, GA promotes leaf growth by regulating cell proliferation and cell expansion. Similarly, brassinosteroid (BR) also facilitates leaf growth via its positive effect on cell proliferation and cell expansion [34, 35]. Meanwhile, cytokinin controls leaf development also via cell proliferation and cell expansion [36, 37].

In crop, leaf usually is flattening, but sometimes, leaves of several varieties are rolled. In rice, rolled leaf could remain leaf upright, reduce shadow, increase planting density, improve photosynthetic efficiency, and increase crop yield [38–40]. *SRL1*, *SLL2*, and *OsHox32* result rolled leaf by regulating bulliform cells [41–43]. *SLL1* and *SRL2* lead rolled leaf through altering structure and processes of sclerenchyma cells [44, 45]. And *ADL1* and *RFS* cause rolled leaf by controlled leaf polarity [46, 47]. For soybean, there are also several researches on rolled leaf. *GmFILa* encodes a YABBY transcription factor, and belongs to FIL subfamily. *GmFILa* showed specific expression patterns in leaf and bud primordia. Ectopic expression of *GmFILa* in *Arabidopsis* altered the partial abaxialization of the adaxial epidermises of leaves, which resulted leaf rolled [48, 48]. *E1*, is known as flowing repressor, which affects soybean flowing and maturity largest, also regulates leaf development. Unifoliolate leaves of *E1-overexpression* lines were smaller and curlier, and which may due to E1 negatively control the expression of *TCP* family genes in soybean [49]. *TCP* family genes play a crucial role in leaf development [50–52]. In soybean, although some genes have been linked to rolled leaf, there is still gap to draw the molecular mechanism that regulates rolled leaf.

To better understand leaf development of soybean, a mutant line with rolled and narrow leaves (named *rl*) was charactered in this study. We analysed the phenotype of *rl*, and using transcriptome analysis revealed the different expression genes between *rl* and wild type HX3. Meanwhile, endogenous hormone analysis and exogenous hormone treatment represented the hormone factors which regulated rolled and narrow leaf in soybean. Our findings contributed to promote the insights into the molecular regulation of leaf morphogenesis in soybean.

## Materials and methods

### Plant materials and measurement of phenotypic traits

In this study, soybean cultivar HX3 (wild-type) and the mutant line *rl* were used. Mutant line *rl* was selected from mutant library which derived from HX3 treated with EMS. First, the seeds of HX3 dipped in water 4 h and dipped in EMS (40 mmol/L) 8 h. After using water flushed seeds several times, the seeds were planted and formed the mutant library. Plants were grown in soil in growth cabinet with 28℃/16 h light and 25℃/8 h dark condition. When plants were flowering, leaf traits were measured by ImageJ. Pod width, pod length and hundred seed weight were measured after pod dried three days.

### Anatomical analysis

Anatomical analysis process was based on others research with some changed [53, 54]. The fully expanded leaflet was sampled from HX3 and *rl* for anatomical analysis. Leaflet was fixed by FAA fixative (70% ethanol: glacial acetic acid: formaldehyde = 89:6:5) for 48 h. Then, samples were dehydrated by 70%, 80%, 90%, and 95% ethanol ordinal, and were treated 30 min every time. Then, samples were dipped in semi-penetrant (ethanol: penetrant = 1:1, penetrant was 100 mL liquid with 1 g HardenerI) overnight at 4℃, and were dipped in penetrant 2–3 h at room temperature. Later, samples were embedded by solution that contained 15 mL penetrant and 1ml HardenerII (Technovit 7100, Heraeus Kulzer). The embedded samples were cut by microtome, the thickness of section was 5 mm. Then the tissue sections were observed under microscope (NE610, Nexcope, Ningbo, China).

### Total RNA extraction and transcriptome sequencing

The third tender leaves which were not unfolded of HX3 and *rl* were collected for RNA extraction and sequencing, and three biological replicates of each sample were used. Trizol Reagent Kit (GENSTONE BIOTECH, Beijing, China) was used to extract RNA. After the content and integrity of RNA reaching the standard, RNA was revers transcription and cDNA libraries were built using VAHTS® Universal V8 RNA-seq Library Prep Kit (Vazyme, Nanjing, China). Then, the cDNA was sequenced by T7 PE150 platform (YINGZI GENE, Wuhan, China).

### Quantitative real-time PCR (qRT-PCR)

RNA was extracted from samples that were used in RNA-seq. After testing content and quality of RNA, first-stand cDNA was synthesized using Evo M-MLV RT Mix Kit with gDNA Clean for qPCR (Accurate Biology, Changsha, China). qRT-PCR analysis was performed using SYBR Green Premix Pro Tag HS qPCR Kit (Accurate Biology, Changsha, China) with the CFX Connnect Real-Time PCR System (Bio-Rad, California, USA). And the primers used for qRT-PCR were showed in Table S2.

### Endogenous hormone content determination

When the third leaves of HX3 and *rl* were not fully unfolded, the leaves were collected, and were stored in liquid nitrogen. Endogenous hormone content was determined by BIOTREE TECH (Shanghai, China). Three biological replicates were prepared.

### Hormone treatment

At seeding stage, 6-BA, IAA, and ABA were used to spraying plants until solution distributed on all leaf surface, spraying H_2_O was the control group. Treatment took one every two days, and remained 10 days. After stopping treatment, leaf traits were observed. 6-BA content was 50 µM, IAA content and ABA content were 10 µM.

## Results

### The difference of leaf morphology between mutate line *rl* and wild-type

**Fig. 1:**
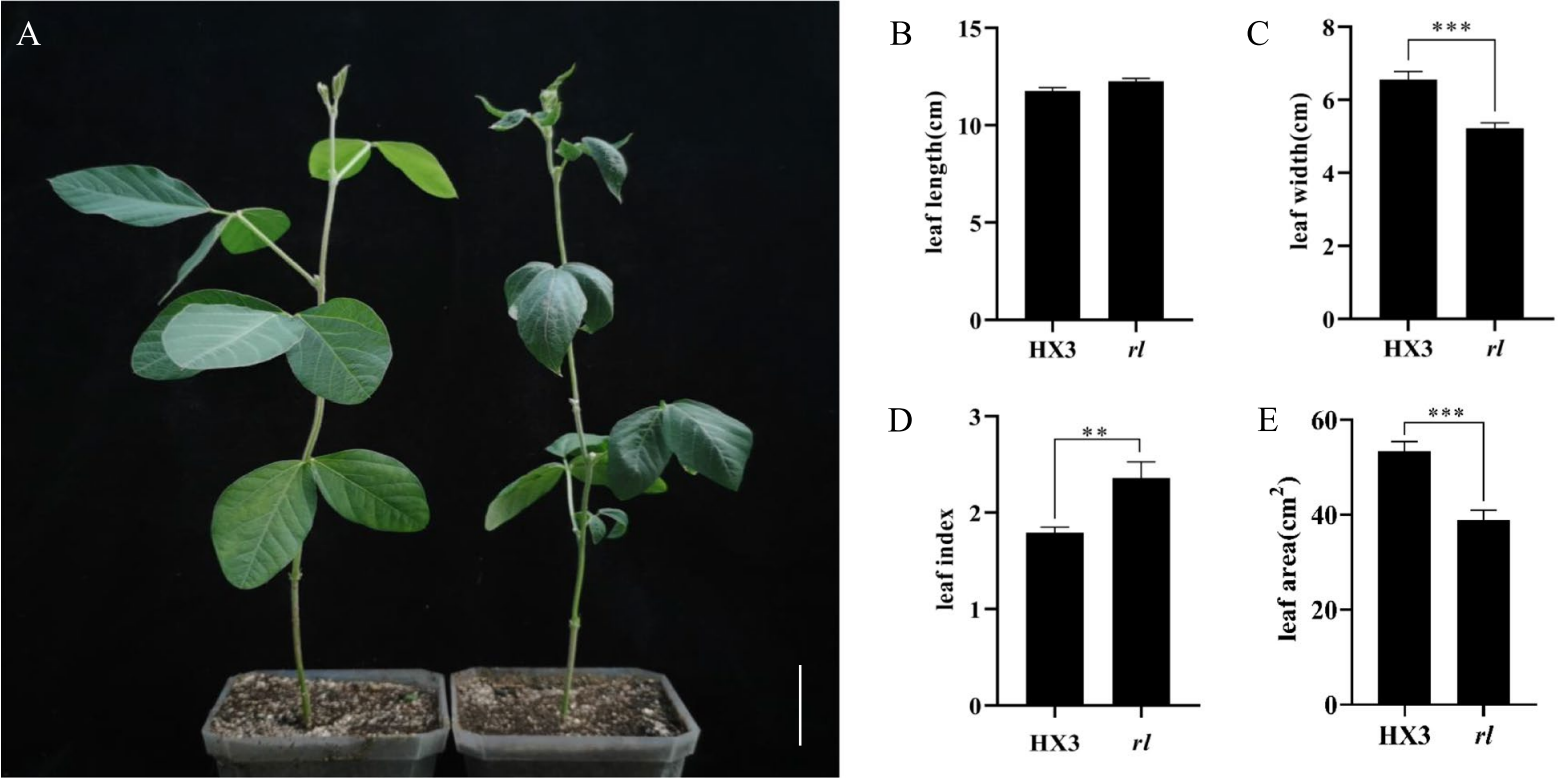
Comparison of plant leaflet between HX3 and *rl*. **A** Phenotype of HX3 (left) and *rl* (right). **B** Leaf length. **C** Leaf width. **D** Leaf index. **E** Leaf area. Data are mean ± SD, *n* = 4. Scale bar represents 5 cm. ‘**’ indicates significant differences at *p* = 0.01 level, ‘***’ indicates significant differences at *p* = 0.001 level

Compared with HX3, the mutant (*rl*) showed apparently rolled and narrow leaf. The leaf of *rl* curled, and was narrower (Fig. 1). Averagely, the leaf width of mutant reduced 23.8% compared with HX3. The leaf length of mutant increased, but didn’t reach significant difference. Therefore, leaf index (the radio of leaf length to width) was increased 44.4% dramatically, and leaf area reduced significantly.

**Fig. 2:**
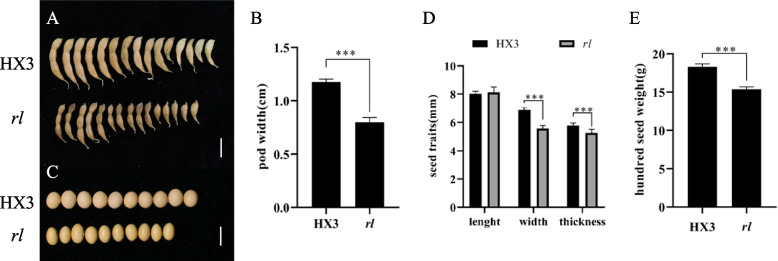
Comparison of pod and seed between HX3 and *rl.* Phenotype of pod (**A**) and seed (**C**) of HX3 and *rl*. **B** Pod width. **D** Seed length, width, and thickness. **E** Hundred seed weight. Data are mean ± SD, *n* = 10. Scale bar represents 1 cm. ‘***’ indicates significant differences at *p* = 0.001 level

The mutant also revealed narrow pod (Fig. 2A, B). By contrast with HX3, the pod width of *rl* was reduced 25%, but the pod length was unchanged. Perhaps, the development of pod affected seeds, seeds of *rl* were also narrower (Fig. 2C, D), and hundred seed weight reduced significantly (Fig. 2E).

### The difference of leaf tissue and cell size between mutate line *rl* and wild-type

**Fig. 3:**
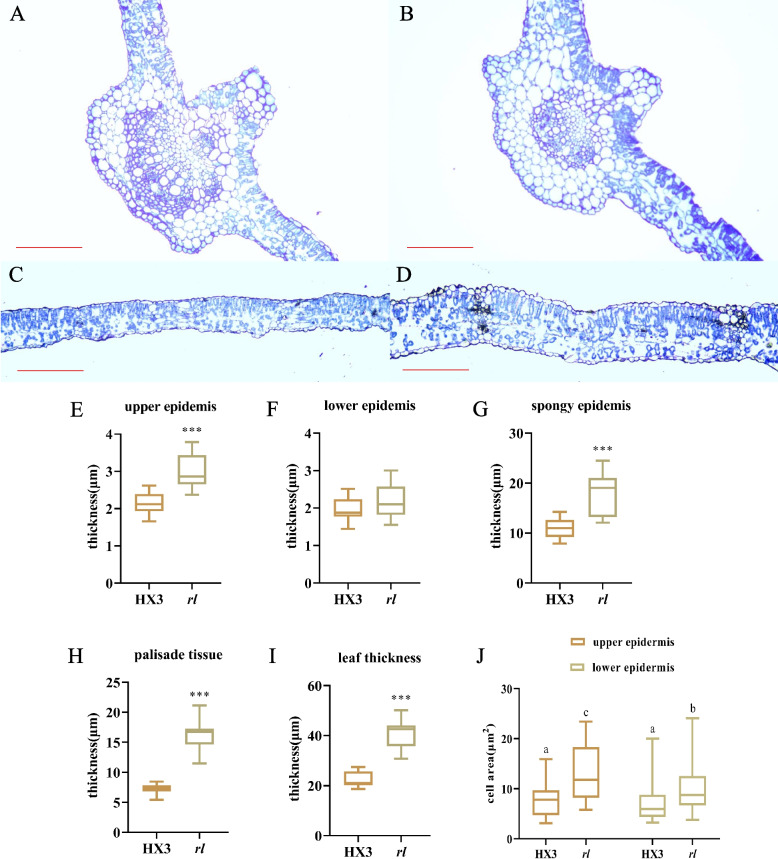
Leaf anatomical structure of HX3 and *rl*. Cross-section of leaf veins of HX3 (**A**) and *rl* (**B**). Blade cross-section of HX3 (**C**) and *rl* (**D**). **E**-**I** Leaf thickness and the thickness of epidermis, palisade tissue and spongy tissue. Data are mean ± SD, *n* = 3. **J** Cell area of epidermis. Data are mean ± SD, *n* = 30. Scale bar represents 50 μm. ‘***’ indicates significant differences at *p* = 0.001 level. Different letters denote significant difference at *p* = 0.05 level

In order to investigate the difference of leaf between HX3 and *rl*, transverse sections of leaflet were used for histological observation (Fig. 3A-D). Compared with HX3, vascular tissue in the main vein of *rl* leaflet was smaller (Fig. 3A, B). Then, using ImageJ analysed leaf cross-section of HX3 and *rl* (Fig. 3C, D). It showed that *rl* leaflet was thicker, because there was thicker upper epidermis, lower epidermis, palisade tissue, and spongy tissue in *rl* (F ig. 3E-I). Though, the differences about thickness of lower epidermis wasn’t significant, the thickness of upper epidermis, palisade tissue, and spongy tissue was strikingly different between HX3 and *rl*. In contrast with HX3, the cell area of upper epidermis and lower epidermis increased significantly in *rl* (Fig. 3J). For HX3, the cell size was similar between upper epidermis and lower epidermis. However, the cell size of upper epidermis was bigger than that of lower epidermis in *rl* (Fig. 3J). Therefore, the difference of cell growth in upper epidermis and lower epidermis may lead rolled leaf of *rl*. Through histological observation, leaflet structure of *rl* changed prominently.

### The differential expression genes between mutate line *rl* and wild-type

**Fig. 4:**
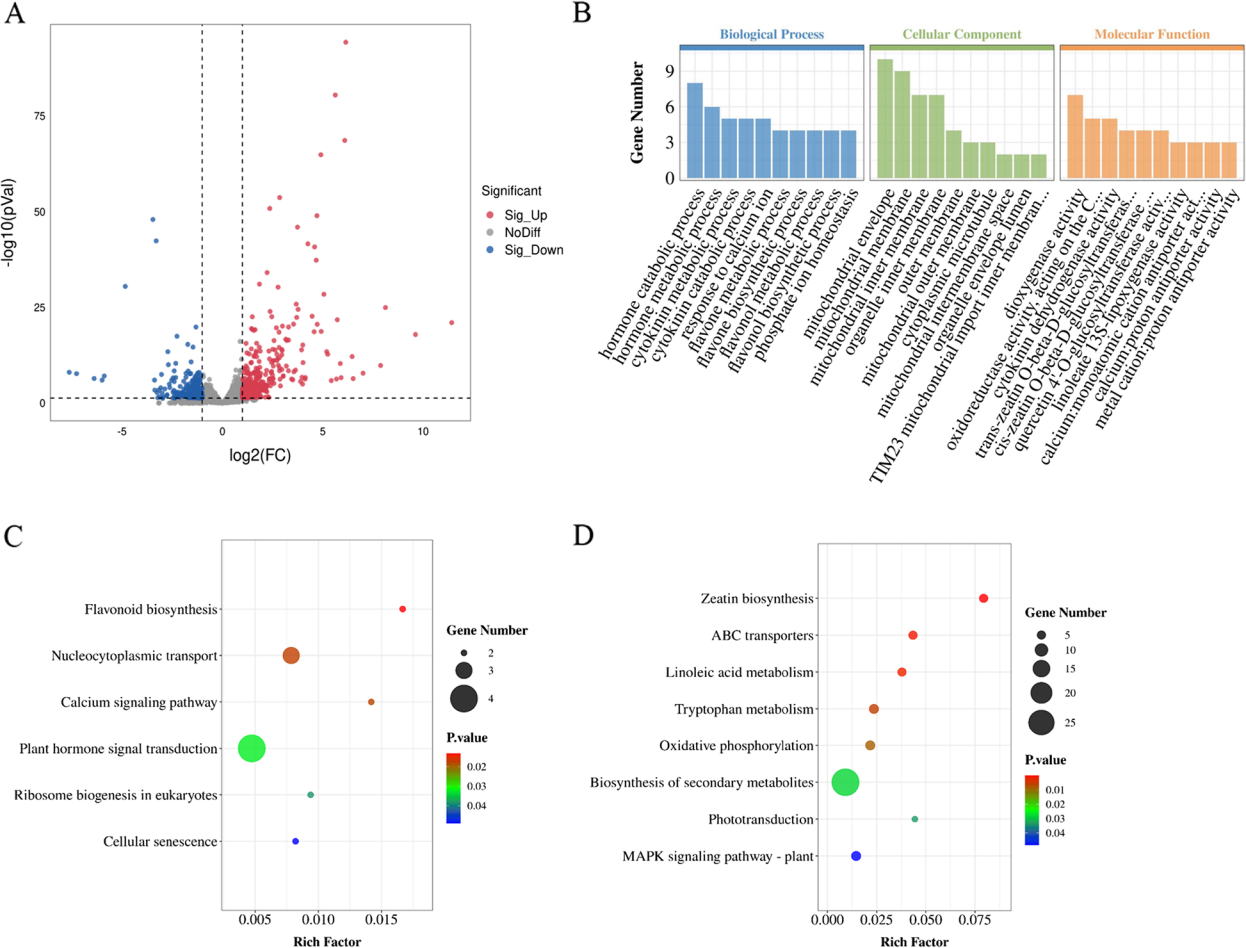
Analysis of different expression genes about HX3 and *rl*. **A** Volcano map of different expression genes. **B** GO analysis of different expression genes. KEGG pathway enriched of down-regulation genes (**C**) and up-regulation genes (**D**)

In order to understand the molecular mechanism that control soybean rolled and narrow leaf, we performed transcriptome analysis. In this study, a total of 360.41 million reads were generated. The mapping ratio of clean reads to reference genome and reference genes were 97.17% and 93.33% in average, respectively (Table S1). All reads located in each region of the transcripts evenly, which indicated that the sequencing data could reveal accurately gene expression level. In all, 357 differentially expressed genes (DEGs) were identified between HX3 and *rl*. Compared with HX3, there were 102 down-regulated genes and 255 up-regulated genes in *rl*, respectively (Fig. 4A).

To explore the function of DEGs between HX3 and *rl*, Gene Ontology (GO) enrichment analysis and Kyoto Encyclopedia of Genes and Genomes (KEGG) pathway analysis were conducted. For GO enrichment analysis, the DEGs were classified into three categories: biological process, cellular component, and molecular function (Fig. 4B). In biological process term, hormone catabolic process, hormone metabolic process, and cytokinin metabolic process were the top three term. For cellular component, the number of DEGs in mitochondrial envelope was largest.

Down-regulation DEGs and up-regulation DEGs were carried out KEGG pathway enrichment respectively, they were classified into different pathway, but were mainly in metabolism class and signaling pathway. “Flavonoid biosynthesis” was the most significantly enrichment pathway, and “Plant hormone signal transduction” had the largest number of genes for down-regulation DEGs (Fig. 4C). While, “Zeatin biosynthesis” was most significantly enrichment pathway and was in up-regulation DEGs only, the largest number of genes appeared in “Biosynthesis of secondary metabolites” class (Fig. 4D). “Zeatin biosynthesis” class was consistent with GO enrichment analysis. Therefore, DEGs related to cytokinin metabolism deserve more attention.

**Fig. 5:**
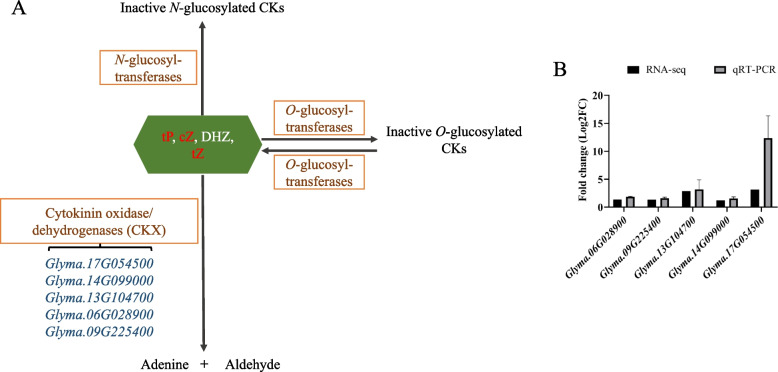
DEGs about cytokinin metabolism pathway. **A** DEGs took part in cytokinin degradation pathway. **B** qRT-PCR validation of the transcriptome data

Several DEGs (*Glyma.06G028900*, *Glyma.09G225400*, *Glyma.13G104700*, *Glyma.14G099000*, and *Glyma.17G054500*) were significantly enriched in cytokinin metabolism. Those genes were up-regulation, which had been validated by qRT-PCR. Those five genes belonged to cytokinin oxidase/dehydrogenase (CKX) genes (Fig. 5). Cytokinin oxidase/dehydrogenase catalyzes the degradation of cytokinin [55]. Therefore, the cytokinin content in *rl* was reduced possibly due to the up-regulation cytokinin oxidase/dehydrogenase genes.

**Fig. 6:**
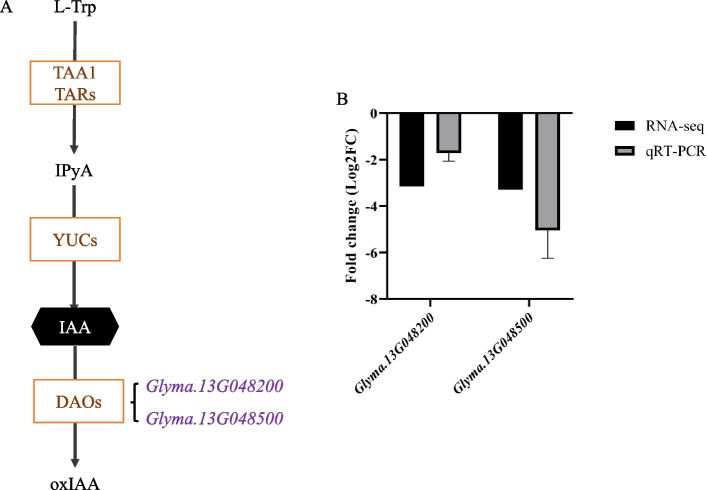
DEGs about auxin metabolism. **A** DEGs took part in auxin degradation pathway. **B** qRT-PCR validation of the transcriptome data

In this study, two DEGs about auxin metabolism were also founded. *Glyma.13G048200* and *Glyma.13G048500* were down-regulation genes, which had been validated by qRT-PCR (Fig. 6). They were orthologs of *AT1G14130* (*DAO1*) that caused IAA degradation [56, 57]. Thus, the IAA content may increase in *rl*.

### The difference of endogenous hormone level between HX3 and *rl*

**Fig. 7:**
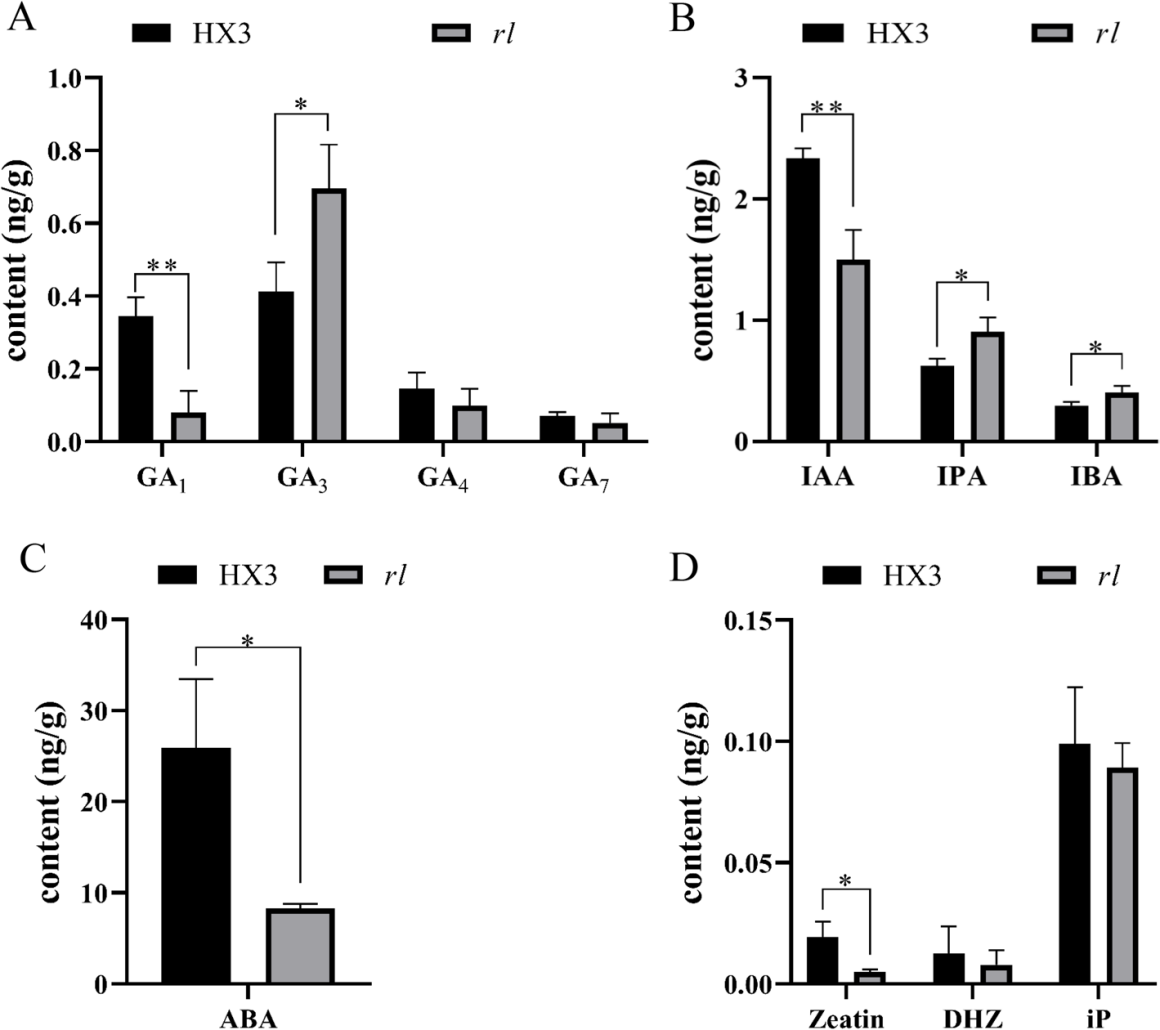
Endogenous hormone content of HX3 and *rl*. **A** The content of gibberellin. **B** The content of auxin. **C** The content of cytokinin. **D** The content of abscisic acid. Data are mean ± SD, *n* = 3. ‘*’ indicates significant differences at *p* = 0.05 level, ‘**’ indicates significant differences at *p* = 0.01 level

For purpose of verifying suppose, endogenous hormone content was measured of HX3 and *rl* (Fig. 7, Table S3). By contrast with HX3, GA_1_ content was lower strikingly in *rl* (Fig. 7A), but GA_3_ content was significantly higher in *rl*, and GA content (GA_1_, GA_3_, GA_4_ and GA_7_) was similar between HX3 (0.9754 ng/g) and *rl* (0.9269 ng/g). IAA content was down-regulation in *rl*, which was opposite of RNA-seq results, and, IBA and IPA content were up-regulation in *rl* (Fig. 7B). ABA content also was significantly lower in *rl* (Fig. 7C). Compared with HX3, zeatin content of *rl* decreased significantly, which was consistent with RNA-seq results (Fig. 7D). Therefore, according to RNA-seq results and endogenous hormone content, cytokinin deserved more attention.

### The effect of exogenous 6-BA, IAA, and ABA for *rl*

**Fig. 8:**
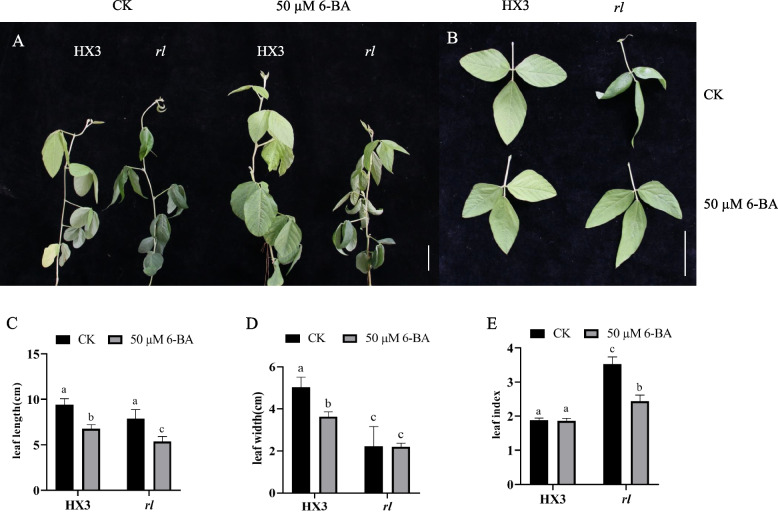
Cytokinin rescued rolled and narrow leaf. Plant phenotype (**A**) and leaf phenotype (**B**) after 6-BA treatment. Leaf length (**C**), leaf width (**D**) and leaf index (**E**) after 6-BA treatment. Data are mean ± SD, n = 3. Scale bar represents 5 cm. Different letters denote significant difference at *p* = 0.05 level

To explore the possible effects of cytokinin on leaf type, using cytokinin 6-BA sprayed HX3 and *rl*. After 6-BA treatment, leaf of *rl* was expanded and not rolled (Fig. 8A, B). Leaf length and leaf width of HX3 reduced significantly all, but leaf index was not changed. Leaf length of *rl* decreased, but leaf width was not changed, thus the leaf index of *rl* decreased significantly. However, the leaf index of *rl* still was larger, and the leaf shape of *rl* was narrower (Fig. 8C-E). Therefore, 6-BA could rescue rolled leaf enough and narrow leaf partly in soybean. After 6-BA treatment, the cell area of new leaf epidermis in HX3 and *rl* were both reduced, and the cell area of *rl* was similar between upper epidermis and lower epidermis, however, the cell area of upper epidermis was bigger than that of lower epidermis without 6-BA treatment (Fig. 3, Fig. S1). Thus, 6-BA rescued leaf type of *rl* through regulating cell development of epidermis. In addition, 6-BA affected only the tender leaves; the mature leaves after treatments were still rolled, demonstrating that the phenotype of rolled and narrow leaves was determined in the early stage of leaf development (Fig. 8A). What’s more, after 6-BA treatment, the expression level of *CKX* genes was both up-regulation in HX3 and *rl* (Fig. S2), thus the expression of those *CKX* genes was induced by 6-BA. However, *CKX* genes increased more greatly in HX3.

**Fig. 9:**
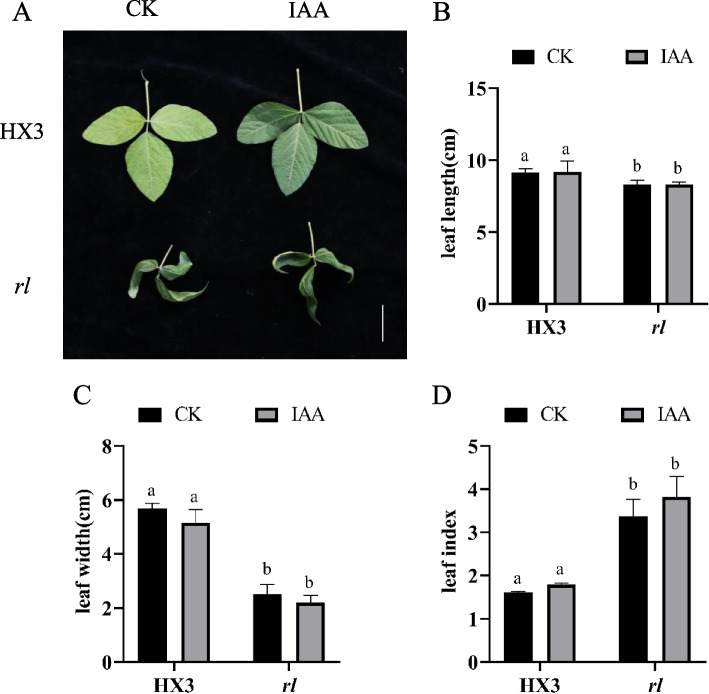
IAA didn’t affect leaf type. **A** Leaf phenotype with IAA treatment. Leaf length (**B**), leaf width (**C**) and leaf index (**D**) with IAA treatment. Data are mean ± SD, *n* = 3. Scale bar represents 5 cm. Different letters denote significant difference at *p* = 0.05 level

In order to test whether lower IAA and lower ABA result in leaf type of *rl*, 10 µM IAA and 10 µM ABA were used to spray HX3 and *rl*. After IAA treatment, leaf was still rolled of *rl* (Fig. 9A). Leaf length didn’t change, leaf width reduced slightly and leaf index increased slightly both of HX3 and *rl* (Fig. 9B-D). After ABA treatment, leaf type of *rl* wasn’t rescued also (Fig, 10). Those results indicated that lower IAA and ABA didn’t affect rolled and narrow leaf type of HX3 and *rl*.

**Fig. 10:**
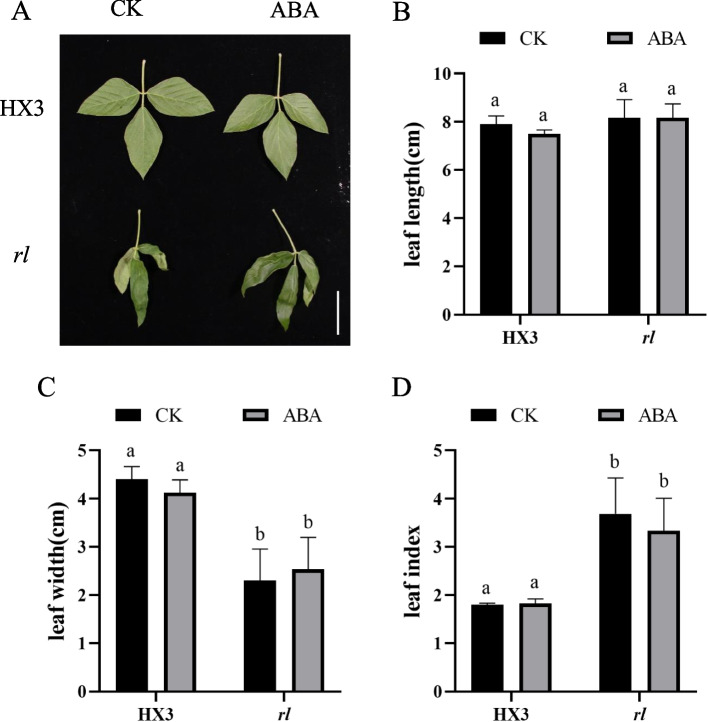
ABA didn’t affect leaf type. **A** Leaf phenotype with ABA treatment. Leaf length (**B**), leaf width (**C**) and leaf index (**D**) with ABA treatment. Data are mean ± SD, *n* = 3. Scale bar represents 5 cm. Different letters denote significant difference at *p* = 0.05 level

## Discussion

Leaves are not only the main organ of photosynthesis, but also determine variation in canopy closure, which controls crop yield [58, 59]. In soybean, canopy coverage is a desirable trait that is a major determinant of yield, and leaflet shape is associated with canopy coverage, which determines distribution of light through the canopy [4]. Therefore, it’s necessary to study the leaflet development of soybean for improving yield.

In soybean, some genes have been identified that regulate leaflet development. Furthermore, several genes control leaf development and grain yield together. *Ln* is known as a crucial gene that controls both leaflet shape and number of seeds per pod [19, 20]. Silencing *GmFAD3* changed leaflet morphology, increased seeds size and enhanced seed yield [60]. Similarly, *GmKIX8-1* knockout lines showed larger leaf size and seed size, 100 seed weight increased [22]. And *BS1* also could lead to similar results. Down-regulation of *BS1* orthologs resulted in increscent organ size and seed weight in soybean [61]. While, overexpression of *GmMYB* reduced leaf area, but increased pod number per plant, seed number per plant and seed weight per plant, hence, *GmMYB* could improve soybean yield [62]. Similar phenomenon also was founded in rice [63]. Therefore, leaf morphology and yield traits may be selected together in the process of domestication. In this study, *rl* also showed narrow pod, narrow seed, and reduced 100 seed weight. Probably, in HX3 and *rl*, a mutation gene resulted rolled and narrow leaf, narrow pod and narrow seed simultaneously. Besides narrow leaflet phenotype, rolled leaflet was another distinct character of *rl*. Few genes have been founded, which control rolled leaflet in soybean, but the molecular mechanism of rolled leaf is still unknown, and the relationship between curly leaflet and yield is still unknown also. Thus, *rl* is a suitable material for further study about rolled leaf. In this study, using RNA-seq data, we analysed the expression level of several genes that were identified to regulate leaf type in soybean. Those 15 genes had similar expression pattern between HX3 and *rl* (Fig. S3). Thus, those 15 genes didn’t contribute leaf type of *rl* under transcriptional level.

Leaf development includes leaf initiation, polarity establishment and maintenance, leaf flattening, and intercalary growth [64], so many genes and hormones participate in this complex process, and cytokinin plays an important role in leaf development. Cytokinin managed SAM maintenance, and inhibited leaf initiation [65], and it acted downstream of *KNOX1*, which was responsible for stem cell maintenance in SAM [26, 66]. Cytokinin oxidase/dehydrogenase (CKX) is the enzyme that catalyzes the irreversible degradation of active cytokinin. Using the lateral organ-specific promoter expressed *AtCKX3* in *Arabidopsis* insulted in smaller leaf and using the *35S* promoter expressed *AtCKX3* in tomato leaded to a strong phenotype of small plants with inhibited growth and small, simplified leaves, which was similar to *phenotype of 35Spro*:*CKX3* in *Arabidopsis* [67, 68]. Lateral research showed the epidermal cell size of *35Spro*:*CKX3* and *ANT*:*CKX3* was increased significantly [69]. In *rl*, the upper epidermal cell size was also increased, and that was consistent with others results. In this study, five genes which encoded cytokinin oxidase/dehydrogenase were identified (Fig. 5). The expression level of these genes was higher in *rl*, which may reduce the level of cytokinin in *rl*. Endogenous cytokinin determination results verified our conjecture, as well. In *rl*, zeatin content was lower, which also revealed those up-regulation *CKX* were related to zeatin degradation mainly in soybean. After 6-BA treatment, those *CKX* genes were up-regulate both in HX3 and *rl* (Fig. S2), this result was consistent others research [70]. But the expression level of *CKX* genes increased more greatly in HX3, which may due to the lower cytokinin level in *rl*. And 6-BA treatment leaded that the cell size of upper epidermis was similar to the cell size of lower epidermis in *rl*, but the cell size of upper epidermis was larger than the cell size of lower epidermis in *rl* without 6-BA treatment. After 6-BA treatment, the cell size of epidermis about HX3 and *rl* both reduced, but the cell size of *rl* was still larger than the cell size of HX3 (Fig. S1, Fig. 3). The leaf phenotype also showed 6-BA could rescue rolled leaflet phenotype enough and rescue narrow leaflet phenotype partially. Therefore, the change of cytokinin content leaded rolled and narrow leaf in *rl* by regulating cell development, but there may be other factors regulate rolled and narrow leaf together with cytokinin, which need more research.

## Conclusions

In this study, we characterized a rolled-leaflet mutant *rl* of soybean. Mutant *rl* showed rolled and narrow leaf, leaf area was smaller. Anatomical and cytological analysis demonstrated that the cell size of epidermis increased observably in *rl*, and cell growth was different between upper epidermis and lower epidermis of *rl*, which may cause rolled and narrow leaf. Transcriptome analysis and endogenous hormone determination showed that up-regulation *CKX* resulted lower cytokinin in *rl*. 6-BA treatment results indicated that cytokinin could rescue rolled and narrow leaflet of *rl* indeed. IAA and ABA were lower in *rl*, but IAA and ABA didn’t affect leaf type in HX3 and *rl*. We speculate that cytokinin is the factor which leaded to rolled and narrow leaf, and plays an important role in leaf development. The results of this study provide information for further understanding of leaf development in soybean.

### Supplementary Information

Supplementary Material 1.

Supplementary Material 2.

## Data Availability

We have uploaded RNA-seq data to NCBI, the accession number is PRJNA1031766.
