# Supplementary material for: Morphological characterization and transcriptome analysis of rolled and narrow leaf mutant in soybean
Source: BMC Plant Biol. 2024 Jul 19;24:686. doi: 10.1186/s12870-024-05389-7 (PMC11264519; doi:10.1186/s12870-024-05389-7)
Supplement: Supplementary file 1 — Supplementary Material 1. [file 12870_2024_5389_MOESM1_ESM.pptx]

## Slide 1
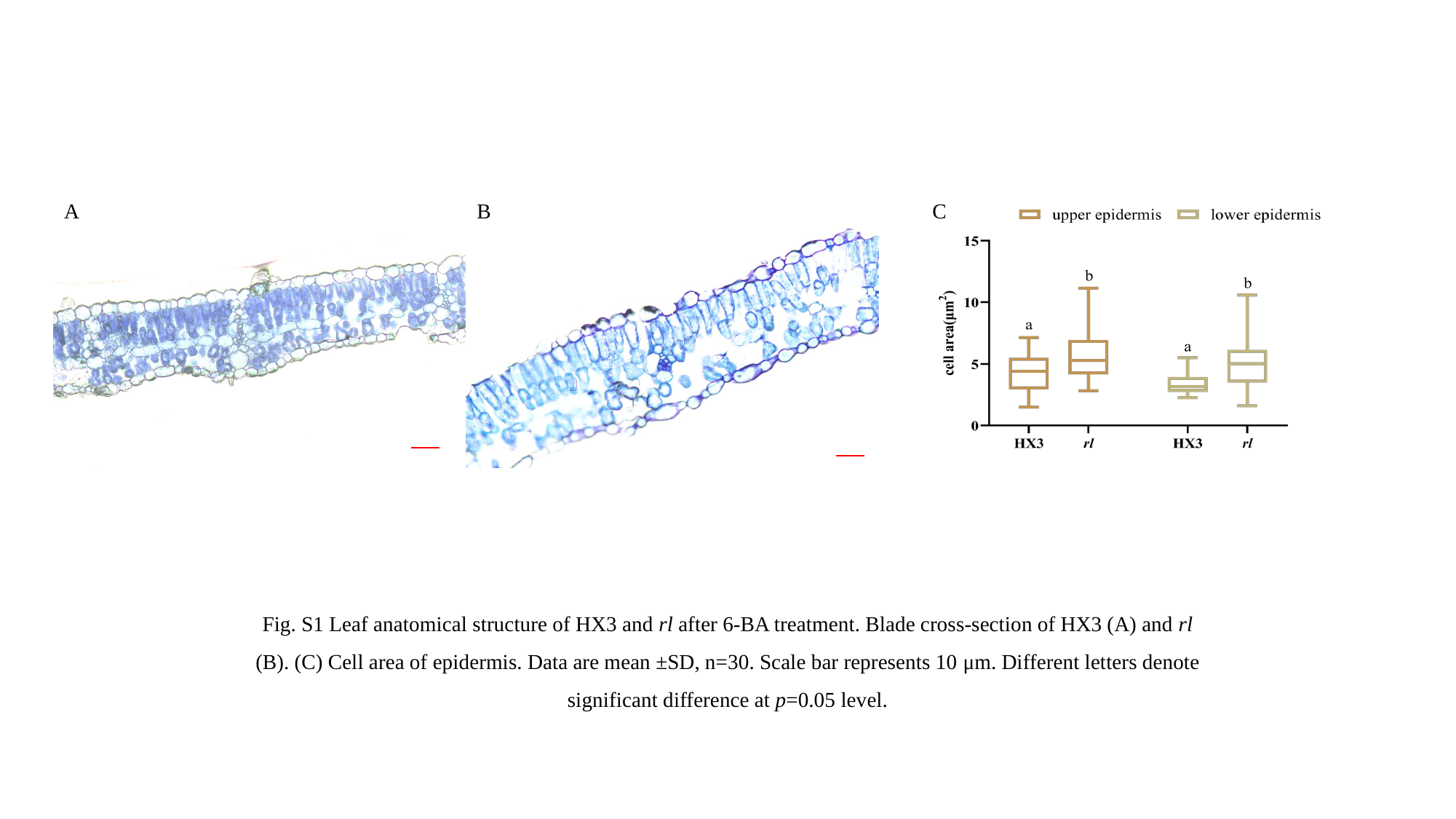

A
B
C
Fig. S1 Leaf anatomical structure of HX3 and rl after 6-BA treatment. Blade cross-section of HX3 (A) and rl (B). (C) Cell area of epidermis. Data are mean ±SD, n=30. Scale bar represents 10 μm. Different letters denote significant difference at p=0.05 level.

## Slide 2
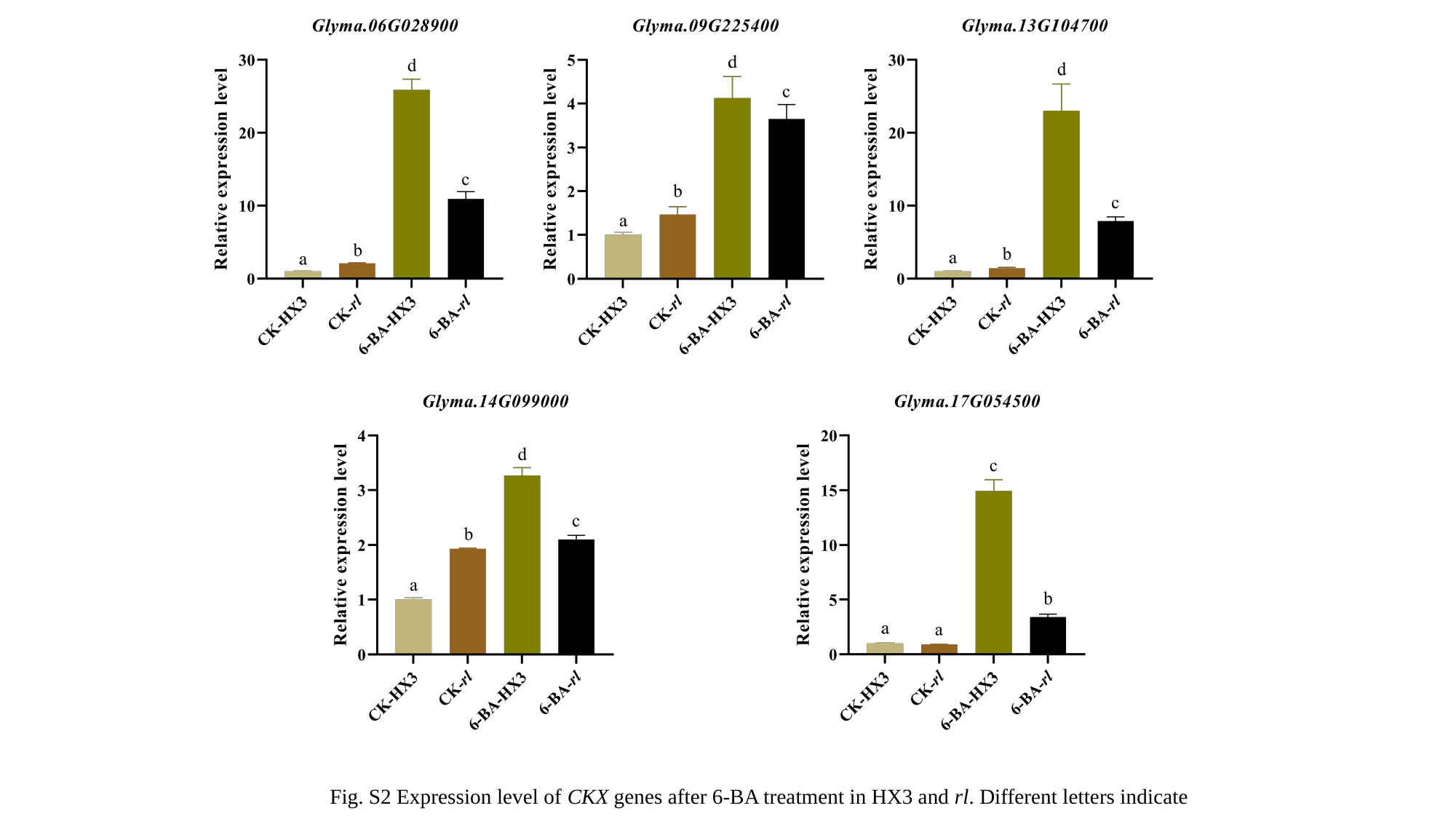

Fig. S2 Expression level of CKX genes after 6-BA treatment in HX3 and rl. Different letters indicate significantly difference at p=0.05 level.

## Slide 3
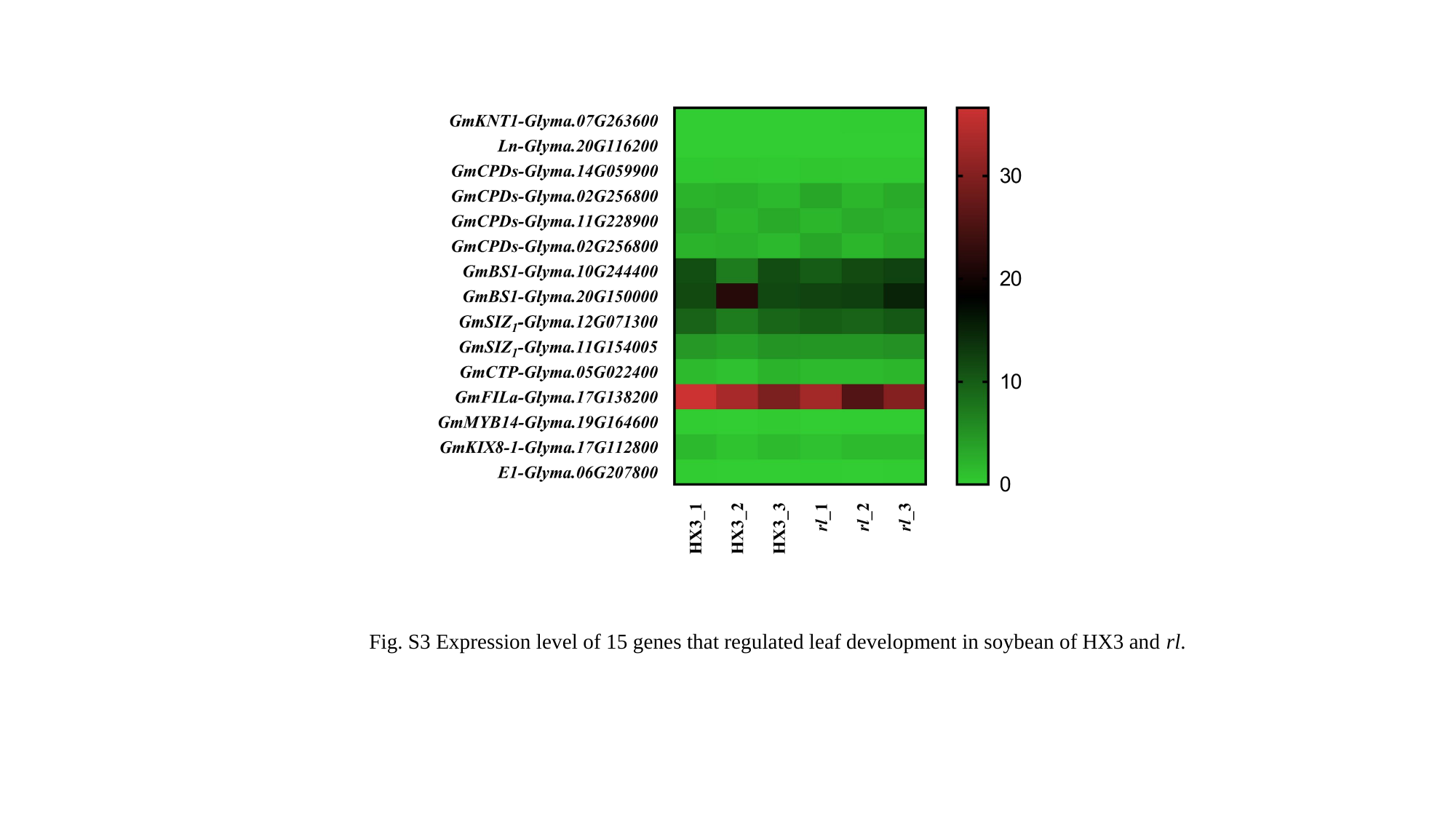

Fig. S3 Expression level of 15 genes that regulated leaf development in soybean of HX3 and rl.
